# Importance of Dietary Phosphorus for Bone Metabolism and Healthy Aging

**DOI:** 10.3390/nu12103001

**Published:** 2020-09-30

**Authors:** Juan Serna, Clemens Bergwitz

**Affiliations:** 1 Yale College, Yale University, New Haven, CT 06511, USA; juan.serna@yale.edu; 2 Section of Endocrinology and Metabolism, Department of Internal Medicine, Yale School of Medicine, New Haven, CT 06519, USA

**Keywords:** dietary phosphorus, inorganic phosphate (P_i_), hypophosphatemia, hyperphosphatemia, mineralization, absorption, paracellular, transcellular

## Abstract

Inorganic phosphate (P_i_) plays a critical function in many tissues of the body: for example, as part of the hydroxyapatite in the skeleton and as a substrate for ATP synthesis. P_i_ is the main source of dietary phosphorus. Reduced bioavailability of P_i_ or excessive losses in the urine causes rickets and osteomalacia. While critical for health in normal amounts, dietary phosphorus is plentiful in the Western diet and is often added to foods as a preservative. This abundance of phosphorus may reduce longevity due to metabolic changes and tissue calcifications. In this review, we examine how dietary phosphorus is absorbed in the gut, current knowledge about P_i_ sensing, and endocrine regulation of P_i_ levels. Moreover, we also examine the roles of P_i_ in different tissues, the consequences of low and high dietary phosphorus in these tissues, and the implications for healthy aging.

## 1. Introduction

Phosphorus is one of the essential elements of the human body and is required for a diverse range of processes, such as ATP synthesis, signal transduction, and bone mineralization. The vast majority (85%) of phosphorus in the body exists as a component of hydroxyapatite [Ca_10_(PO_4_)_6_(OH)_2_] in the extracellular matrix of bone and teeth [1]. In contrast, intracellular phosphorus accounts for 14% of total body phosphorus, and only 1% is present, mostly as inorganic phosphate (P_i_), in extracellular fluids [1]. Phosphorus most commonly occurs as a salt of phosphoric acid, which is an essential physiological buffer referred to as P_i_. Although we will focus on this form of phosphorus, it is important to note that phosphorus is also a component of phospholipids, DNA, RNA, ATP, and creatine phosphate (CrP). At physiological pH, P_i_ is apportioned 4:1 between its divalent form, HPO_4_^2−^, and its monovalent form, H_2_PO_4_^−^, respectively [2]. Moreover, P_i_ also forms dimers (such as pyrophosphate) and polymers (such as polyphosphate) [3,4,5]. Alternatively, P_i_ may be covalently bound in organic molecules, including inositol pyrophosphates, membrane phospholipids, phosphoproteins, and ribonucleic acids [3,4,5].

As a result of its importance in health, the maintenance of extracellular P_i_ homeostasis is imperative. Chronic P_i_ deficiency can result in both bone loss through resorption [6,7] and contribute to myopathy [8] and frailty. Moreover, severe acute hypophosphatemia may cause cardiac and respiratory failure, leading to death [9]. On the other hand, high extracellular P_i_ is similarly associated with adverse health outcomes, including coronary artery calcification, worsening renal function, premature aging, and increased mortality [10,11,12,13].

The nutritional environment of Western cultures is, among other features, notable for its very high phosphorus content. This is in no small measure because P_i_ salts are routinely added to processed foods for a variety of reasons, including taste and food preservation. The dysregulation of extracellular P_i_ is implicated in skeletal disorders as well as vascular calcification in chronic kidney disease and cardiovascular disease [14,15]. This review will examine how dietary phosphorus is absorbed by the body (with an emphasis on recent insights about endocrine regulation of P_i_ homeostasis) and the effects of dietary phosphorus as a nutrient in various organ systems. Additionally, we will examine the effects of dietary phosphorus in longevity and how possible adverse effects may indicate a need for closer examination of the use of P_i_ salts as additives in Western foods. We will highlight areas still poorly understood—for example, the function of P_i_ transporters in dental health, cardiovascular health, and the nature and molecular basis of paracellular P_i_ absorption in the gut.

## 2. Phosphate Absorption from the Diet in the Gut

The concentrations of phosphorus in sea water or soil are in the micromolar range [16]. Accordingly, the normal situation for unicellular organisms, plants, and certain aqueous animals is that of phosphorus deprivation, for which the uptake and sensing of P_i_ are stimulated by default. It is only when P_i_ is available in higher quantities that this uptake and sensing system is turned off [17]. In higher species (such as fish and mammals), the reverse is the case, since these species can move to seek food to meet their nutrient needs. Particularly for terrestrial mammals and humans, dietary phosphorus is plentiful, and therefore, homeostatic processes that prevent phosphorus intoxication have evolved.

**Figure 1:**
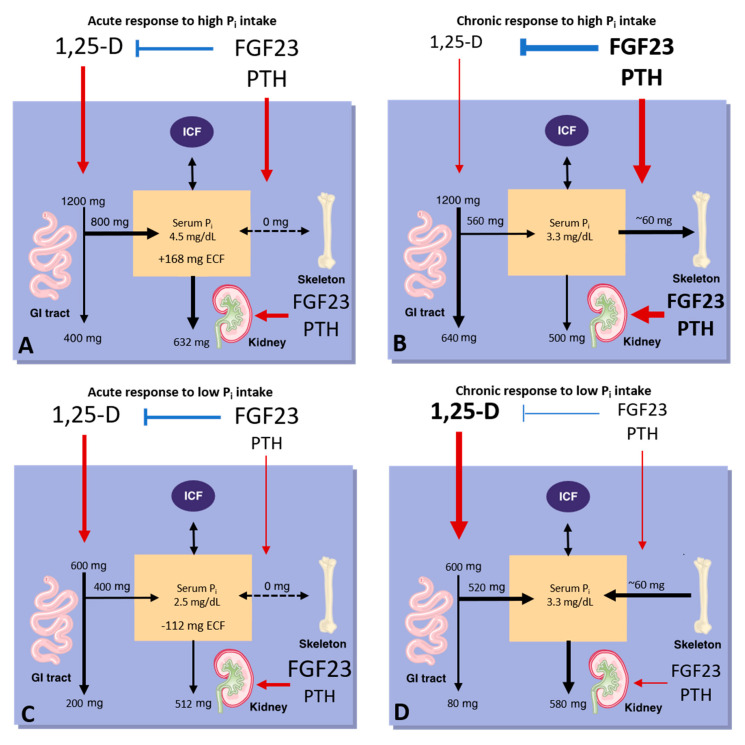
Phosphate homeostasis. (**A**) The acute response and (**B**) the chronic response to increases in phosphate intake. (**C**) The acute response and (**D**) the chronic response to decreases in phosphate intake. Details are provided in the text. Numbers show hypothetical shifts of phosphorus between body compartments for a 70 kg adult based on [28]. GI, gastrointestinal. ICF, intracellular fluid. PTH, parathyroid hormone. 1,25(OH)_2_D, 1,25-dihydroxycholecalciferol (calcitriol). FGF23, fibroblast growth factor 23. P_i_, inorganic phosphate. dL, deciliter. mg, milligram. SI conversion: 1 mg phosphorus = 0.32 mmol phosphorus. Adapted from [29].

In Western diets, phosphorus (usually as a P_i_ salt) is frequently used as an additive in processed foods [18,19]. Recent estimates indicate that phosphorus intakes often exceed the recommended daily allowance (RDA) by one-and-a-half to two-fold [20]; in adults between the ages of 19 and 70, this corresponds to an intake that ranges between 1500 and 1700 mg/day for men and 1000 and 1200 mg/day for women [20]. Intestinal P_i_ absorption is highest in infancy and childhood and declines with age [21]. However, it remains robust at approximately 50–70% of bioavailable phosphorus [21]. Of this ingested phosphorus, 16 mg/kg/day is unidirectionally absorbed in the proximal intestine, and 3 mg/kg/day is lost through endogenous pancreatic, bile, and gut secretions [22]. The result is a net absorption of 13 mg/kg/day of phosphorus, which can enter the extracellular fluid and be utilized by tissues such as bone (Figure 1) [22]. The majority of dietary phosphorus is absorbed as P_i_ in the small bowel by two pathways: a passive paracellular pathway and a transcellular absorption pathway. In conditions of abundant dietary P_i_ intake, 70% of intestinal P_i_ uptake occurs primarily by passive paracellular diffusion, while only 30% occurs via sodium (Na^+^)-dependent, carrier-mediated transcellular transport [21,23,24,25,26]. However, in experimental animals, it is estimated that the two pathways contribute roughly equally to intestinal P_i_ absorption [27].

### 2.1. Paracellular Phosphate Absorption Pathway

In the paracellular absorption pathway, P_i_ moves from the diet into the circulation passively through tight junction complexes [30]. However, the specific molecular identities of the components associated with paracellular P_i_ absorption have not been identified. Dietary P_i_ absorption through the paracellular pathway occurs primarily in the small intestine, and to a lesser extent in the colon [31,32]. The permeability for P_i_ in these intestinal segments is similar to Na^+^ [31,32]. Although both monovalent and divalent P_i_ exist under physiological conditions, monovalent P_i_ is slightly preferred for paracellular P_i_ absorption [31]. Although earlier estimates suggest that the paracellular pathway is the major mechanism for P_i_ absorption, it is important to note that this pathway is low-affinity. As such, it is sufficient only when phosphorus is present in the diet in high concentrations. The paracellular pathway is also non-saturable, allowing for P_i_ absorption in potentially toxic quantities. Paracellular P_i_ absorption is susceptible to chemical inhibitors, but it is not known to be regulated by hormonal factors (see Section 3, Endocrine regulation of phosphate homeostasis). It is likewise unknown whether paracellular transport is unidirectional or whether it can result in the ‘leaking’ of P_i_ from the extracellular space into the gut under certain conditions. 

### 2.2. Transcellular Absorption Pathway/Transporter-Mediated Phosphate Absorption

The second pathway for intestinal P_i_ absorption is mediated primarily by the sodium-dependent phosphate transport protein 2b, NPT2b (encoded by *SLC34A2*). To a lesser extent, this transcellular transport is also mediated by the type 3 sodium-dependent transporters, PIT1 (encoded by *SLC20A1*) and PIT2 (encoded by *SLC20A2*), although the role of the latter is not clear [21,33]. This high affinity and saturable transporter-mediated pathway is present in the duodenum and jejunum [34,35,36,37], is stimulated by 1,25-dihydroxyvitamin D [1,25(OH)_2_D, or calcitriol], and accounts for 30% of intestinal P_i_ absorption when phosphorus is abundant in the diet [21,34,38,39].

NPT2b uses the transmembranous Na^+^ gradient to transport P_i_ into cells against its own electrochemical gradient at a stoichiometry of 3:1 Na^+^:P_i_ [40]. Both murine [40,41] and swine [42] Npt2b preferentially transport divalent P_i_. The apparent Michaelis–Menten affinity constant (K_m_) for murine Npt2b for P_i_ is 10 µM at −60 mV, as determined via kinetic characterization in *Xenopus* oocytes [40,41]. This preferential transport of divalent P_i_ also explains why P_i_ uptake via swine NPT2b expressed in *Xenopus* oocytes increases with alkaline pH levels and is maximal at pH 8.5 [42].

## 3. Endocrine Regulation of Phosphate Homeostasis

The normal blood concentration of P_i_ in humans is 2.5–4.5 mg/dL and is regulated through the control of intestinal absorption of P_i_ from the diet, by the release of P_i_ from intracellular stores acutely and from bone remodeling chronically, and renal excretion (reviewed by [14,43,44,45]). This homeostasis is maintained by parathyroid hormone (PTH), fibroblast growth factor 23 (FGF23), calcitriol, and other factors discussed below. In turn, P_i_ feeds back to regulate the secretion of these hormones. This process is often referred to as endocrine P_i_ sensing [46] but is still poorly understood (for several excellent reviews, see [46,47,48]).

### 3.1. Clinical Chemistry of Phosphate

Clinical laboratories use differing methods to either measure P_i_ (colorimetric assays) or phosphorus (flame photometry). However, P_i_ measurements are converted to phosphorus (1 mg/dL P_i_ contains 0.32 mmol/L phosphate, which is equal to 0.32 mmol/L phosphorus). Additionally, it is important to collect fasting samples to determine serum P_i_ levels, since feeding causes hyperinsulinemia, which reduces serum P_i_ levels by inducing intracellular shifts of P_i_ [49]. Moreover, it is also important to consider that the serum concentration of phosphorus follows a circadian rhythm in addition to that modulated by dietary phosphorus; serum phosphorus levels vary throughout the day and are lowest in the early morning [50]. Currently, analysis of serum P_i_ is not a routine measurement in clinical practice; given the potential for adverse health effects caused by high levels of dietary phosphorus and serum P_i_ (discussed later in this review), and the high prevalence of P_i_ additives in Western foods, it may be necessary to reevaluate the importance of such testing.

### 3.2. Regulation of Phosphate Absorption in the Gut

#### 3.2.1. Calcitriol

1,25(OH)_2_D, or calcitriol, is the active form of vitamin D and the key hormone that regulates transcellular P_i_ absorption in the intestine by stimulating the expression of NPT2b. A deficiency of P_i_ upregulates calcitriol [6], which will be discussed further in Section 4.3 below. Calcitriol binds to its nuclear hormone receptor vitamin D_3_ receptor (VDR) [51]. This calcitriol/VDR complex forms nuclear heterodimers with retinoic acid X-receptor (RXR), which then can bind the vitamin D-responsive elements of target genes [51] to regulate gene expression [52,53,54]. Thereby, calcitriol promotes intestinal P_i_ absorption both directly by inducing NPT2b expression in the gut and indirectly by increasing calcium absorption, thereby improving P_i_ absorption by preventing the formation of insoluble CaP_i_ in the gut lumen [54,55].

#### 3.2.2. Phosphorus Depletion

Similar to 1,25(OH)_2_D, dietary phosphorus depletion is considered one of the predominant physiological stimuli of intestinal P_i_ absorption [25,39,56]. Chronic adaptation to a low-phosphate diet in wild-type (WT) rats appears to go along with the upregulation of *Npt2b* in the jejunum and upregulation of *Npt2a* in the proximal tubules, where the bulk of filtered P_i_ is reabsorbed [25,37,57,58,59]. However, it appears that low-P_i_-diet-induced Npt2b upregulation is independent of the 1,25(OH)_2_D–VDR axis [60]. This was shown in mice that had a normal upregulation of Npt2b by a low-P_i_ diet despite being VDR- and 1-α hydroxylase-deficient [60]. Additionally, low dietary phosphorus increases the activity of NPT2b by post-transcriptional mechanisms [18,21] and mobilizes P_i_ from the bone mineral via increased resorption [7].

#### 3.2.3. Estrogen

Similar to calcitriol, estrogen increases Na^+^-dependent P_i_ absorption in the gut. In response to estrogen treatment, Xu et al. showed increased brush border membrane vesicle (BBMV) P_i_ uptake accompanied by an increased abundance of Npt2b protein in rat intestine [61]. Further, estrogen treatment also increases *Npt2b* mRNA levels in rats, suggesting transcriptional upregulation of the gene encoding Npt2b in response to estrogen [61]. A similar result was found using human intestinal epithelial (Caco-2) cells [61].

#### 3.2.4. Glucorticoids

Glucocorticoids (GCs) inhibit P_i_ uptake in the intestine. Methylprednisolone injection in suckling animals resulted in a 3–4-fold reduction of NPT2b protein and mRNA levels, which reduced Na^+^-P_i_ uptake [62]. This suggests a possible regulatory role for GCs at the transcriptional level. However, other work done demonstrated that GC injection resulted in increased fucosyl transferase activity in suckling rat intestine [62,63]. This finding suggests a role for corticoids in intestinal maturation and the regulation of NPT2b glycosylation in a non-genomic fashion [62,63].

#### 3.2.5. Epidermal Growth Factor

Epidermal growth factor (EGF) similarly inhibits intestinal P_i_ absorption. EGF decreased promoter activity in Caco-2 cells transfected with human *NPT2b* promoter constructs and also decreased *NPT2b* mRNA abundance in both Caco-2 and rat intestinal cells by 40–50%, indicating that EGF transcriptionally downregulates *NPT2b* [64]. The molecular basis for this effect is that EGF reduces the binding affinity of the v-myb avian myeloblastosis viral oncogene homolog (c-myb) for the EGF responsive element in the *NPT2b* gene promoter [65], resulting in decreased promoter activity and therefore reduced *NPT2b* mRNA abundance [65]. Additionally, Xu et al. found that this regulatory effect involves EGF receptor-mediated activation of the mitogen-activated protein kinase (MAPK), protein kinase C (PKC), and protein kinase A (PKA) pathways [65].

## 4. Regulation of Systemic Phosphate Homeostasis

### 4.1. PTH

PTH is a peptide of 84 amino acids that is secreted by the parathyroid glands and signals through the PTH and PTH-related protein receptor [(also known as parathyroid hormone receptor 1 (PTHR1)], which is expressed in osteoblasts, osteocytes, chondrocytes, and proximal tubular cells [46]. P_i_ stimulates the secretion of PTH in the parathyroids, which in turn stimulates the synthesis of calcitriol in the proximal tubules and thereby indirectly stimulates intestinal P_i_ absorption [66]. Additionally, PTH stimulates bone turnover, resulting in the release of P_i_ from the skeleton [67,68]. However, the net effect of PTH is to lower blood levels of P_i_ because PTH also reduces the stability of the type II Na^+^-P_i_ co-transporters (NPT2a and NPT2c) at the renal brush border membrane, which reduces the reabsorption of P_i_ from the urine [69,70]. This process is mediated by Na^+^/H^+^ exchange regulatory cofactor 1 (NHERF-1), which exists as a complex with NPT2a at the apical membrane of the proximal tubular cells [69,71]. NHERF-1 is phosphorylated via the cyclic adenosine monophosphate-PKA and phospholipase C-PKC signal transduction pathways following the activation of PTHR1 [21,69,71].

### 4.2. FGF23

FGF23 is a member of the fibroblast growth factor (FGF) family that is produced by osteocytes and osteoblasts in the skeleton [72,73,74], and it stimulates P_i_ excretion in the kidneys [75]. This most recently identified physiologic regulator of renal P_i_ excretion [76] provides a mechanism by which skeletal mineral demands can be communicated to the kidney. Thus, the skeleton can influence the P_i_ economy of the entire organism through FGF23. Dietary P_i_, particularly when present in excess amounts, stimulates the synthesis of FGF23 [77]. In turn, FGF23 reduces the expression of NPT2a and NPT2c and thereby reduces P_i_ reabsorption [78]. This process requires binding of the C-terminal tail of FGF23 to the klotho 1 and klotho 2 domains of α-Klotho (KL, which functions as a co-receptor) [79]. Additionally, binding of the FGF-like N-terminal domain to isoform c of FGF receptor 1 (FGFR1c) is also required [79]. Dimerization of this FGF23-KL-FGFR1c heterotrimer by heparan sulfate [79] is required for the activation of the extracellular signal-regulated kinases 1 and 2 (ERK1/2) [80]. Then, activation of ERK1/2 results in NHERF-1 dependent internalization of NPT2a and NPT2c from the apical membrane of proximal tubular cells in the kidneys [81]. Additionally, FGF23 reduces the expression of *CYP27B1* (encoding CYP27B1, the 25-hydroxy-vitamin D 1-α hydroxylase that synthesizes calcitriol) and stimulates the expression of *CYP24A1* (encoding CYP24A1, the vitamin D 24-hydroxylase that degrades calcitriol) [82,83]. As such, FGF23 inhibits the synthesis of calcitriol [82,83]. The net effect of this NPT2a/c internalization and decreased circulating calcitriol levels is lower blood levels of P_i_.

### 4.3. Calcitriol

Calcitriol is the active metabolite of vitamin D as mentioned above. Its synthesis by CYP27B1 and degradation by CYP24A1 are regulated by PTH, FGF23, calcium, and P_i_ [54]. PTH stimulates calcitriol synthesis by inducing the expression of CYP27B1 and by suppressing the expression of CYP24A1 [54]. FGF23 decreases calcitriol levels by suppressing the expression of CYP27B1 and stimulating the expression of CYP24A1 [46,84]. In turn, calcitriol increases FGF23 levels, forming a negative feedback loop. The actions of P_i_ and calcium on calcitriol synthesis are predominantly mediated by FGF23 and PTH, respectively [85,86,87,88,89]. Additionally, calcitriol inhibits PTH both directly by transcriptionally repressing the gene encoding PTH and indirectly by upregulating the calcium-sensing receptor (CASR, a protein that modulates PTH secretion by calcium in the parathyroid cells) [51]. Calcitriol, as mentioned already above, stimulates NPT2b expression in the intestine and thereby increases both the absorption of P_i_ in the gut and consequently circulating blood P_i_ levels [55]. Since it suppresses PTH, calcitriol also indirectly reduces P_i_ excretion in the kidneys. Therefore, the net effect of calcitriol is to increase blood P_i_.

## 5. Disorders of Phosphate Homeostasis

In this review, we will focus on disorders of intestinal P_i_ absorption, which can be divided into disorders of intake, bioaccessibility, bioavailability, and regulatory hormones. While generally acquired, some of these disorders have underlying genetic causes (as reviewed below). If left untreated, dietary phosphorus insufficiency can result in rickets or osteomalacia [90,91]. Furthermore, a prolonged reduction of serum P_i_ levels <1.0 mg/dL can result in several more health issues, including rhabdomyolysis, cardiac muscle dysfunction with congestive heart failure, and leukocyte dysfunction, among other abnormalities [92,93,94]. Excess blood P_i_ can lead to tissue calcifications and excess syndromes caused by hyperparathyroidism and high circulating FGF23 levels. The effects of dietary phosphorus deficiency or excess will be discussed in detail for each tissue in the next chapters. 

### 5.1. Phosphorus Content in the Western Diet

Phosphorus is abundant in the Western diet. The RDA for phosphorus from the diet is 700 mg/day [20]. However, most Americans far exceed this recommendation [20], since a lack of mandated reporting on nutrition facts labels causes phosphorus content to be commonly unidentified on American food labels [95]. Phosphorus additives tend to be common in prepared frozen foods, dry mixes, packaged meats, bread and baked goods, and some yogurts (where P_i_ additives may be added in addition to the phosphorus contributed by milk) [96]. These additives mainly consist of phosphoric acid, phosphates, or polyphosphates [97], and they were found on average to add 736 mg more phosphorus/day compared to additive-free diets [96]. Even in balanced 2200 kcal/day diets, phosphorus consumption as averaged over four days totaled 1677 ± 167 mg/day in additive-enhanced diets [98]. In low-additive diets designed to meet U.S. Department of Agriculture recommended guidelines for fat, protein, carbohydrate, and phosphorus intake, the averaged total was 1070 ± 58 mg phosphorus/day [98]. When separately evaluated by gender, males aged 19–70 have an average daily phosphorus intake that ranges between 1500 and 1700 mg, and women aged 19–70 usually consume between 1000 and 1200 mg phosphorus/day [20].

Similarly, phosphorus intake in Europe averages 1000–1767 mg/day for both sexes [99]. This elevated intake may also be due to unclear phosphorus labeling since, in Europe, information about phosphorus is generally only available in the ingredients list [100]. However, even in this case, phosphorus may be listed as different P_i_ additives or identified only by E number (a code assigned to food additives in Europe) [100].

National Health and Nutrition Examination Survey data demonstrated an association between high dietary phosphorus intake (>1400 mg/day) and all-cause mortality in U.S. adults after adjusting for other known contributors [101]. This threshold of 1400 mg/day is routinely exceeded by men aged 14–71 in the U.S., suggesting a negative effect of high dietary phosphorus on human longevity [20].

In addition to attention to labeling and phosphorus content in the diet, physicians need to be aware that therapeutic phosphorus preparations often list the mass of the phosphate salt, which includes oxygen, sodium, and potassium. Therefore, phosphorus content varies for the specific preparation being prescribed, and this should be considered in consultation with the pharmacist and hospital formulary (Table 1) [21].

### 5.2. Influence of Dietary Components and Drugs on the Bioaccessibility of Phosphate

Of course, dietary phosphorus deficiency leads to a total body P_i_ deficiency. Furthermore, several dietary components can have a pronounced effect on the bioaccessibility of P_i_. When combined with an insulin-mediated cellular change in P_i_ during refeeding, total body P_i_ deficiency causes hypophosphatemia (defined as serum P_i_ levels below 2.5 mg/dL, and it is considered severe at levels below 1.5 mg/dL) [50,103]. Refeeding syndrome can cause severe hypophosphatemia in malnourished individuals with alcoholism as well in intensive care or institutionalized individuals [49]. However, hypophosphatemia is rare in the general population, since phosphorus is ubiquitous in the Western diet (as already mentioned above) [98]. In turn, high dietary phosphorus intakes as high as 4000 mg/day result in only minor increases in serum P_i_ concentrations due to the high efficiency of renal excretion [104], provided that ingestion is spread throughout the day. However, undiluted cow’s milk can provide sufficiently high P_i_ to induce hyperphosphatemia in infants [15]. Moreover, bowel preparations that use P_i_-containing laxatives (such as oral phosphosodas) can result in severe hyperphosphatemia and in some cases renal failure due to nephrocalcinosis [105,106].

pH is also an important factor controlling the bioaccessibility of P_i_ from the diet in the gut. At pH values above 6.5, dibasic P_i_ predominates but has low solubility (30 mg/dL) [107,108]. At pH values below 6.5, monobasic P_i_ predominates with a much higher solubility (1800 mg/dL) [107,108]. In a study of infants who were fed an elemental diet and exhibited unexplained hypophosphatemia, a commonality between many children was treatment with gastric proton pump inhibitors (PPIs) [109]. The effect of this treatment would have likely been abnormally high gastric pH [109]. Given that P_i_ solubility decreases with increasing pH (particularly above the acidic range), it is possible that increased gastric pH could have resulted in reduced mineral absorption [108,109]. However, it should be noted that the authors did not identify systemic acid–base abnormalities in these children, and P_i_ status was corrected with alternative Pi salts rather than an alteration of acid-modifying medications [109]. Similarly, alkaline pH inhibits P_i_ transport in rat intestinal BBMVs [110]. Conversely, acidic pH increases P_i_ uptake in rat intestinal BBMVs, and this might be partially mediated by an unidentified, Na^+^-coupled P_i_ transporter that prefers monovalent P_i_ [110].

Certain antacids, such as magnesium–aluminum hydroxides, sucralfate, and calcium-containing antacids, can also reduce the bioaccessibility of P_i_ [111,112]. These antacids reduce P_i_ bioaccessibility by binding to dietary P_i_ and by forming insoluble P_i_ salts in the small intestine that prevent absorption [111,112]. Thus, prolonged use of P_i_-binding antacids can cause hypophosphatemia [111].

Phytate, which is the major form of phosphorus in the seeds of plants, is a compound that can form indigestible mineral phytate salts in humans (who lack the enzyme phytase that can release P_i_ from phytate) [113]. At physiological pH, phytate binds calcium with high affinity [114]. If the calcium concentration is high, phytate forms indigestible, multiple calcium phytate salts [113]. However, phytate becomes digestible at low calcium concentrations due to reduced calcium binding [113]. Kim et al. demonstrated that high phytate/low calcium diets increase intestinal P_i_ absorption in rats following intestinal phytate hydrolysis that makes P_i_ bioaccesible [113]. This P_i_ load results in secondary hyperparathyroidism and renal P_i_ wasting, which appears to be independent of FGF23 [113]. Calcium supplementation alleviates this effect [113]. Therefore, attention to dietary calcium may be especially important in vegetarians or other individuals who consume high-phytate diets to avoid hyperphosphatemia and its associated effects.

### 5.3. Influence of Dietary Components, Drugs, and Disorders on the Bioavailability of Phosphate

There exist a variety of molecules containing a P_i_ moiety that can act as competitive inhibitors of NPT2b and thereby inhibit intestinal P_i_ transport. Phosphonoformate (PFA, a phosphonocarboxylate) has a K_i_ value of 0.37 mM in rat small intestine BBMVs, which indicates a low affinity of NPT2b for PFA as compared to P_i_ [115]. 2′-Phosphophloretin (2′-PP), a derivative of the plant chalcone phloretin, more strongly inhibits Na^+^-dependent P_i_ uptake, with K_i_ values of 38 ± 7 nM in rabbit intestinal BBMVs and 42 ± 8 nM in rat intestinal BBMVs [116]. Pentavalent arsenate can be transported by NPT2b and also acts as a competitive inhibitor of NPT2b (K_i_ = 51 µM for rat Npt2b in *Xenopus* oocytes) [115]. Of course, the inhibition of intestinal P_i_ transport would reduce P_i_ bioavailability.

On the other hand, nicotinamide adenine dinucleotide (NAD) acts as a non-competitive inhibitor of intestinal P_i_ transport in vivo [117]. Similarly, triazole derivatives also act in a non-competitive fashion, inhibiting up to 61% of intestinal P_i_ absorption as measured by in vivo experiments [118,119]. Finally, although tenapanor is not a direct inhibitor of NPT2b, repeat administration of tenapanor reduces transcellular P_i_ absorption by decreasing the apical membrane expression of NPT2b at the small intestine [120]. This effect occurs at the transcriptional level [120]. These molecules and their effects on intestinal P_i_ absorption are described in further detail in several excellent reviews [31,33,118].

There are no known competitive inhibitors of paracellular P_i_ absorption in the intestine, which relies on passive diffusion and, differently from transporter-mediated absorption, is non-saturable. However, a notable non-competitive inhibitor of paracellular P_i_ transport is Tenapanor. Tenapanor increases transepithelial electrical resistance (TEER) of the sodium/hydrogen exchanger isoform 3 (NHE3, encoded by SLC9A3) [120]. This increase in TEER reduces tight junction permeability to P_i_ and therefore reduces intestinal P_i_ absorption [120]. Since paracellular P_i_ absorption is the major mechanism by which P_i_ is absorbed when it is abundant in the diet, drugs that target paracellular rather than transcellular P_i_ absorption could be as effective at preventing hyperphosphatemia in conditions such as chronic kidney disease (CKD) as P_i_ binders. The development of such drugs could be aided by the identification of P_i_-specific occludins and claudins. Additionally, it would be interesting to know if leaky gut, whereby P_i_ is lost through the paracellular route, can occur in humans (perhaps in situations where drugs or other causes reduce the concentration of P_i_ in the intestinal lumen such that P_i_ flows outward into the lumen).

Vitamin D intoxication caused by acute doses of vitamin D >10,000 international units (IU)/day and potentially chronically through extended administration of doses >4000 IU/day [121,122] increases intestinal P_i_ absorption via the upregulation of NPT2b [54,55] and also increases bone resorption [7,123]. Both factors can contribute to the development of hyperphosphatemia. Additionally, high levels of calcitriol inhibit the production of PTH, thereby blunting PTH-mediated removal of NPT2a/c from the renal proximal tubules [66,124]. This effect contributes to hyperphosphatemia due to reduced renal P_i_ excretion [66,124]. Conversely, vitamin D deficiency causes decreased intestinal P_i_ absorption, resulting in rickets or osteomalacia as well as secondary hyperparathyroidism [125,126].

Finally, certain diseases and conditions can also affect P_i_ bioavailability. Inflammatory bowel diseases are often characterized by chronic diarrhea [127] and result in the malabsorption of P_i_ [128]. Conversely, metabolic acidosis causes increased NPT2b abundance in mouse small intestinal BBMVs, although this increase is not accompanied by a corresponding increase in *NPT2b* mRNA [129]. As a result, metabolic acidosis increases P_i_ uptake, potentially to buffer acid equivalents and contribute to the restoration of acid–base homeostasis [129].

Acquired disorders of P_i_ homeostasis are summarized below in Table 2.

### 5.4. Genetic Disorders of Intestinal Phosphate Absorption

In addition to acquired disorders, there are several genetic disorders of intestinal P_i_ absorption that concern the active, transcellular transport of P_i_. Thus far, no genetic abnormalities affecting the passive, paracellular transport of P_i_ have been reported. 

The key protein involved in active intestinal P_i_ transport is NPT2b [35]. NPT2b deletion is embryonic lethal in mice, and homozygous loss of function (LOF) mutations of NPT2b in humans cause pulmonary alveolar and testicular microlithiasis [151,152]. On the other hand, genetic disorders of vitamin D synthesis or action can impair intestinal P_i_ absorption. Hereditary 1,25(OH)_2_D-resistant rickets (HVDDR) is characterized by mutation in the VDR gene, which results in vitamin D resistance [153]. As a result of this vitamin D resistance, HVDDR symptoms include hypophosphatemia, hypocalcemia, secondary hyperparathyroidism, and severe rickets with osteomalacia [153]. Similarly, vitamin D-resistant rickets type 1A is characterized by mutation in *CYP27B1* [154]. Thus, this disorder causes calcitriol deficiency, which results in hypophosphatemia, hypocalcemia, and rickets [15,154].

Although LOF mutations in claudins and occludins that affect the paracellular transport of other minerals (such as magnesium) have been described, no disorders caused by LOF mutations in P_i_-specific tight junction proteins have been reported thus far.

### 5.5. Other Disorders of Phosphate Homeostasis

**Figure 2:**
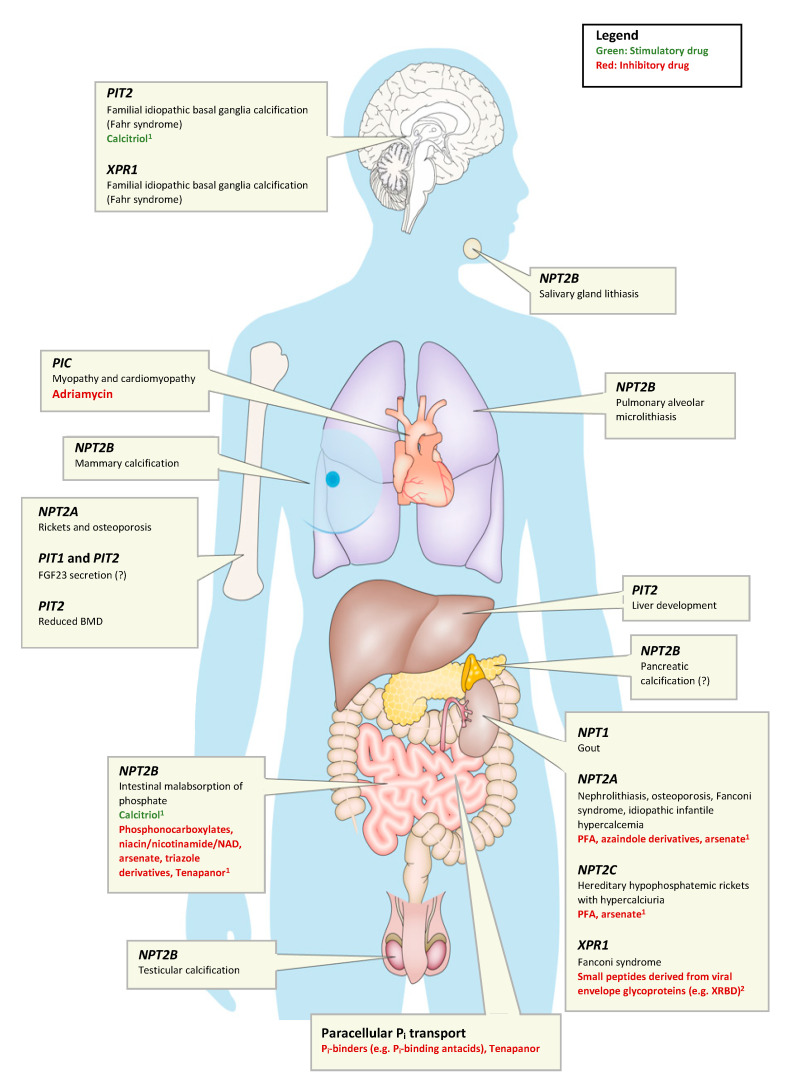
Human disorders of phosphate homeostasis caused by transporters of inorganic phosphate (P_i_). Compounds that inhibit P_i_ transport are denoted in red, while compounds that stimulate P_i_ transport are denoted in green. ^1^ These compounds may affect their respective P_i_ transporters in tissues other than the ones that the compounds are listed under. For example, NPT2A inhibitors may also affect P_i_ transport in the bone. Additionally, tenapanor is not a direct inhibitor of P_i_ transport through NPT2B. **^2^** XPR1 small peptide inhibitors were only reported in *in vitro* studies. Question mark indicates unknown. FGF23, fibroblast growth factor 23. BMD, bone mineral density. NAD, nicotinamide adenine dinucleotide. *NPT1*, sodium-dependent phosphate transport protein 1. *NPT2A*, sodium-dependent phosphate transport protein 2A. *NPT2B*, sodium-dependent phosphate transport protein 2B. *NPT2C*, sodium-dependent phosphate transport protein 2C. PFA, phosphonoformic acid. *PIT1*, type III sodium-dependent P_i_ transporter 1. *PIT2*, type III sodium-dependent P_i_ transporter 2. *PIC*, (*SLC25A3*, solute carrier family 25 member 3). *XPR1*, xenotropic and polytropic retrovirus receptor 1. XRBD, soluble ligand that can bind XPR1. Adapted from [46].

For the large number of disorders of P_i_ homeostasis that do not concern P_i_ as a nutrient (listed in Table 3 and visually in Figure 2), the reader is referred to several excellent recent reviews [15,21,155]. These disorders can be broadly divided into disorders of extracellular P_i_ homeostasis or disorders of intracellular P_i_ homeostasis. Disorders of extracellular P_i_ homeostasis can be further categorized as being FGF23-dependent, PTH-dependent, and FGF23 or PTH-independent. This distinction is mainly helpful diagnostically, since P_i_ absorption and calcium absorption are differentially affected (Table 3).

## 6. Metabolic Phosphate Sensing

Before discussing the tissue-specific roles of P_i_, it may be helpful to summarize what is known about metabolic P_i_ sensing. For several excellent reviews as well, see [48,192,193]. Recent evidence suggests that the type 3 Na^+^-dependent P_i_ transporters PIT1 and PIT2 have an important role in metabolic P_i_ sensing. Interestingly, PIT1 and PIT2 might sense extracellular P_i_ without requiring its translocation, which is a process that is also referred to as transport-independent P_i_ sensing [194,195]. Therefore, these transporters may serve as sensors for extracellular P_i_ in addition to regulating intracellular P_i_ levels. The transport-independent P_i_-sensing process might involve co-receptors: for example, FGFR1 [48] and the CASR [196]. It is also possible that P_i_ directly binds and inhibits the CASR [196,197]. Finally, there is recent evidence that intracellular P_i_ stimulates the synthesis of 5-diphosphoinositol 1,2,3,4,6-pentakisphosphate (IP7) or 1,5-bisdiphosphoinositol 1,2,3,4-tetrakisphosphate (IP8), which are molecules that signal cellular P_i_ sufficiency and stimulate P_i_ efflux via *Xenotropic and polytropic retrovirus receptor 1* (*XPR1*) [198,199,200,201]. Metabolic P_i_ sensing has a role separate from the endocrine P_i_ sensing discussed above, and it is important for maintaining intracellular P_i_ concentrations and producing intracellular effects such as gene activation, as is discussed in more detail below. 

### 6.1. Extracellular Phosphate Sensing

Extracellular P_i_ activates the mitogen-activated protein kinases ERK1 and ERK2 (encoded by *MAPK1* and *MAPK3*) in most cell types [202]. This process is evolutionarily conserved between *Drosophila melanogaster* and humans [202]. ERK1/2 activation is blocked by pharmacological or genetic inhibitors of the type 3 Na^+^-dependent P_i_ transporters [203]. Since multiple type 3 transporters can fulfill this role, it might be intracellular P_i_ that is sensed to activate ERK1 and ERK2.

**Figure 3:**
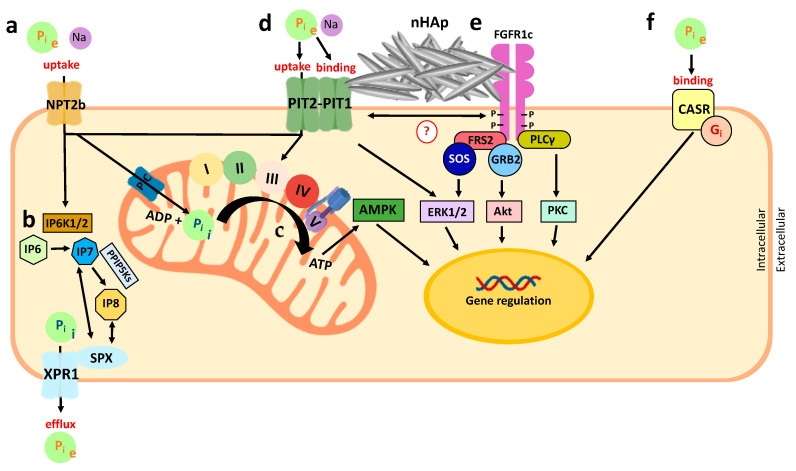
Schematic representation of metabolic P_i_ sensing in mammals (modified from [193]). P_i_ sensing in mammals can be divided into the distinct processes of extracellular P_i_ (P_i_ e) sensing and intracellular P_i_ (P_i_ i) sensing. (**a**) Extracellular P_i_ can be imported into the cell by the sodium-dependent phosphate transport protein 2b (Npt2b), which changes the intracellular P_i_ concentration. (**b**) An increase in intracellular P_i_ stimulates synthesis of 5-diphosphoinositol 1,2,3,4,6-pentakisphosphate (IP7) from inositol hexakisphosphate (IP6) by the inositol hexakisphosphate kinases 1 and -2 [199,200]. IP7 can be further converted into 1,5-bisdiphosphoinositol 1,2,3,4-tetrakisphosphate (IP8) by diphosphoinositol pentakisphosphate kinases [201]. P_i_ efflux through xenotropic and polytropic retrovirus receptor 1 (XPR1) maintains the intracellular P_i_ concentration, and this process is stimulated by the binding of IP7 and IP8 to the SPX domain of this P_i_ exporter [200,201]. (**c**) In addition to stimulating IP7 and IP8 synthesis, P_i_ can also stimulate ATP flux by serving as a substrate for ATP synthesis at complex V of the respiratory chain in the mitochondria and by stimulating the respiratory chain directly [216,217]. ATP inhibits the AMP-activated protein kinase (AMPK) pathway, while AMP and ADP activate it [218,219]. (**d**) PIT1 and PIT2, similar to Npt2b, function as P_i_ transporters, which raise intracellular P_i_. PIT1 and PIT2 also heterodimerize in response to P_i_ and activate the extracellular signal-regulated kinases 1 and 2 (ERK1/2) pathway transport-independently [204,220]. (**e**) The binding of nanohydroxyapatite (nHAp) crystals to the cell surface may bridge PIT1 and FGFR1 [206]. FGFR1 activates the AKT, protein kinase C (PKC), and ERK1/2 pathways. (**f**) In addition to PIT1, PIT2 and FGFR1, the calcium-sensing receptor (CASR) may also function as an extracellular P_i_ sensor, at least in parathyroid cells [196,197]. P_i_ acts at arginine residue 62 of the CASR as a non-competitive antagonist [196,197], thereby inhibiting the inhibitory G protein G_i_ [221]. Through the actions of AMPK, AKT, PKC, ERK1/2, and G protein, extracellular P_i_ can regulate gene transcription, such as the expression of osteopontin in bone cells [18,206,222,223] and vascular smooth muscle cells [224,225]. P_i_, inorganic phosphate. P_i_ e, extracellular P_i_. P_i_ i, intracellular P_i_. Na, sodium. NPT2b, sodium-dependent phosphate transport protein 2b. IP6K1/2, inositol hexakisphosphate kinases 1 and -2. IP6, inositol hexakisphosphate. IP7, 5-diphosphoinositol 1,2,3,4,6-pentakisphosphate. IP8, 1,5-bisdiphosphoinositol 1,2,3,4-tetrakisphosphate. XPR1, xenotropic and polytropic retrovirus receptor 1. SPX, a domain of XPR1. PIC, mitochondrial phosphate carrier. ADP, adenosine diphosphate. ATP, adenosine triphosphate. AMPK, adenosine monophosphate-activated protein kinase. PIT1, type III sodium-dependent P_i_ transporter 1. PIT2, type III sodium-dependent P_i_ transporter 2. nHAp, nanohydroxyapatite. FGFR1c, fibroblast growth factor receptor 1 isoform c. FRS2, FGFR substrate 2. PLCγ, phospholipase C gamma isoform. SOS, son of sevenless. GRB2, growth factor receptor bound protein 2. ERK1/2, extracellular signal-regulated kinases 1 and 2. AKT, protein kinase B. PKC, protein kinase C. CASR, calcium-sensing receptor. G_i_, inhibitory G protein. (I, II, III, IV, V), complexes I-V of the mitochondrial respiratory chain.

However, several observations suggest that extracellular P_i_ is sensed in the cell membrane. Using green fluorescent protein-tagged versions of PIT1 and PIT2, these transporters were shown to dimerize in response to P_i_, which in turn activates ERK1/2 (Figure 3) [204]. This activation appears to occur even if amino acid mutations that block P_i_ transport are introduced into PIT1 and PIT2 [194]. However, it is currently unclear which downstream molecules mediate the activation of ERK1/2 when these transporters function as ‘transceptors’.

The exposure of murine bone marrow stromal cells (BMSCs), which are cells with osteoblastic potential, to nanohydroxyapatite (nHAp) crystals increases the expression of *osteopontin* (*Opn*) and reduces the expression of *alkaline phosphatase* (*Alp*) in a dose-dependent manner [205]. These authors further showed that nHAp activates FGFR substrate 2 (FRS2) and ERK1/2 signaling downstream of Pit and Fgfr, and the inhibition of ERK1/2 blocks the regulation of *Opn* gene expression [206]. Likewise, pharmacologic inhibition of either Pit or Fgfr in BMSCs decreased the expression of *Opn* and derepressed *Alp* [206]. Electron microscopic evaluation showed nHAp at the cell surface of BMSCs, suggesting that nHAp signaling occurs without the internalization of nHAp [206]. Co-localization of immunostaining for Pit, Fgfr, and nHAp further confirmed that nHAp may bridge Pit and Fgfrs in the membrane of BMSCs, and this may be important for mediating the biological effect of nHAp (Figure 3) [206]. 

Additional evidence that FGFR1 functions as a P_i_ sensor was provided in UMR106 rat osteosarcoma cells. Exposure of these cells to high extracellular P_i_ causes the autophosphorylation of FGFR1 at multiple tyrosine residues [207], although it is currently unclear whether P_i_ binds directly to FGFR1. The sequential phosphorylation of six FGFR1 tyrosine residues (653, 583, 463, 766, 585, and 654) leads to the activation of FGFR1 signaling [208,209], which involves the phosphorylation of FRS2 and ERK1/2, and the gene expression of Early growth response 1 (*Egr1*), ETS variant 4, and ETS variant 5 (*Etv5*) [48,207]. The expression of EGR1 and ETV5 upregulates the polypeptide N-acetylgalactosaminyltransferase 3 (GALNT3) [48,207], although this upregulation may require other transcription factors [48]. GALNT3 is the enzyme required for O-glycosylation of FGF23 at threonine 178, whereby it stabilizes bioactive iFGF23 [48].

Lastly, crystallographic studies showed the binding of P_i_ to the CASR [196,197]. The CASR is highly expressed in the parathyroid glands and distal convoluted renal tubules, and it inhibits PTH secretion by the parathyroids and the reabsorption of calcium from the urine upon the binding of calcium [210]. Conversely, P_i_ inhibits CASR in a non-competitive fashion, resulting in the stimulation of PTH secretion by parathyroid cells [197]. It is unclear at the moment whether the CASR also regulates FGF23 secretion and bioactivity.

### 6.2. Intracellular Phosphate Sensing

Upon uptake via NPT2b and PIT1/2, intracellular (IC) P_i_ stimulates the inositol hexakisphosphate kinases 1 and 2 (IP6K1 and -2) and synthesis of the second messenger IP7 from inositol hexakisphosphate (IP6) [199,200]. IP7 is further converted into IP8 by diphosphoinositol pentakisphosphate kinases (PPIP5Ks) [201]. The binding of IP7 or IP8 to the SPX domain of XPR1 [198,199] triggers P_i_ efflux from the cell, regulating the IC P_i_ concentration [200,201]. 

Cells overexpressing PIT2 showed a concomitant efflux in response to the resulting increase of P_i_ uptake, possibly to maintain IC ATP and P_i_ levels [211]. The P_i_ efflux depends on IP7/IP8 signaling, which promotes efflux through XPR1 [211] and is absent in *XPR1* KO cells or when IP6Ks are blocked pharmacologically [211]. Thus, IP7/IP8 may be important for intracellular P_i_ homeostasis controlled by PIT2 and XPR1 [211].

Subcellular compartments might further sequester intracellular P_i_. The mitochondrial P_i_ carrier protein (PIC, encoded by *SLC25A3*) is part of the multiprotein complex that makes up the mitochondrial permeability transition pore (mPTP) [46]. The mPTP regulates mitochondrial membrane potential and mitochondrial apoptosis [212] and is important for skeletal and cardiac muscle function [213]. PIT1 localizes to the endoplasmic reticulum (ER), where it seems to be involved in regulating the ER stress of growth plate chondrocytes [214]. Finally, large and small conductance chloride channels transport P_i_ into the sarcoplasmic reticulum of rabbit skeletal muscle [215]. However, whether these compartments participate as intracellular sensors for P_i_ is currently unknown.

Since the cellular uptake of P_i_ lowers extracellular P_i_, the sensing of intracellular P_i_ (metabolic P_i_ sensing) is likely independent of extracellular P_i_ sensing (endocrine P_i_ sensing). However, it is possible that endocrine sensing activates intracellular P_i_ sensing pathways in endocrine cells. 

## 7. Importance of Dietary Phosphorus for Bone Health

### 7.1. General Importance of Phosphate for Bone Health

P_i_ is required for proper plate growth and bone development, and along with calcium, it comprises the hydroxyapatite that is deposited during mineralization of the vertebrate skeleton. As a result, P_i_ is critical for the mineralization process (particularly during the growth spurt at puberty [226]), to maintain bone strength after the closure of the epiphyses [227], and during fracture repair and remodeling [228]. The process of matrix mineralization requires the secretion of matrix vesicles (MVs) by osteoblasts and hypertrophic chondrocytes [229,230]. The phosphatase PHOSPHO1 can liberate P_i_ from phosphocholine and other lipids in the MV membrane [21]. P_i_ is also thought to be imported into the MVs via PIT1 [21]. MVs induce hydroxyapatite crystal formation [231]. In the presence of sufficient concentrations of extracellular calcium and P_i_, these crystals continue to grow after the dissolution of the MV membrane [231]. The ambient extracellular P_i_ concentration in bone is maintained by tissue non-specific alkaline phosphatase (TNAP), which is abundant in MVs [21]. TNAP cleaves pyrophosphate (PP_i_) and other organic bisphosphonates, which generates two P_i_ molecules [21]. A high P_i_/PP_i_ ratio is generally thought to favor mineralization [232,233,234]. Clinically relevant hypophosphatemic individuals exhibit an increased activity of alkaline phosphatase [22,235]. This allows bone-specific alkaline phosphatase activity to serve as a marker of P_i_ homeostasis in the bone [22,235].

Dietary phosphorus deprivation impairs cell metabolism and causes skeletal demineralization to occur. Moreover, secondary changes due to the adaptive hormonal response (i.e., upregulation of calcitriol, suppression of PTH and FGF23) can be observed. The main process that stimulates bone resorption is calcitriol-mediated activation of osteoclasts through the receptor activator of NF-κB (RANK)–RANK Ligand (RANKL) signaling, as described in more detail below [236,237]. This process is more important with prolonged dietary phosphorus deficiency and can cause rickets and stunted growth in children and osteomalacia in adults [90,91]. In addition, there are several acute effects of phosphorus deprivation, which will be described separately for each tissue below. 

Similarly, high dietary phosphorus intake adversely affects bone health. Firstly, high dietary phosphorus can reduce calcium absorption and serum calcium concentrations through the formation of insoluble calcium–P_i_ complexes [238]. This reduction in serum calcium causes reduced calcium binding to CASR and thereby reduces the inhibition of CASR [239]. Recently, it was shown that P_i_ can bind and directly inhibit CASR [196,197]. As a result, secondary hyperparathyroidism develops, which in turn stimulates bone resorption [197,239,240]. Additionally, P_i_ has direct effects on bone cells: for example, P_i_ stimulates the expression of bone matrix protein osteopontin, which is a mineralization inhibitor [18,222]. Thereby, high dietary phosphorus is associated with increased risk for bone fractures, as shown in a study of 2420 Brazilian individuals in whom every 100 mg of dietary phosphorus intake increased the risk of fracture by 9% [241]. Recent insights suggest that FGF23 (whose levels are determined by P_i_ concentrations) may impair bone matrix mineralization independently of calcium by transcriptionally suppressing TNAP (although this effect also suppresses OPN) [242,243]. High phosphate containing soft drinks may finally affect dental mineralization, as discussed later in this review.

### 7.2. Role of Phosphate in Chondrocytes

Chondrocytes produce and maintain the extracellular matrix of joint cartilage and permit the longitudinal growth of long bones through endochondral ossification. P_i_ is essential for normal hypertrophic differentiation and apoptosis, which was shown in several primary [244,245,246] and stable chondrocytic cell lines [247,248]. Hypertrophic differentiation and apoptosis require the activation of ERK1/2 and the mitochondrial–caspase-9 pathway [244]. These processes are blocked by ablation or pharmacological inhibition of the PIT1 transporter or of the mitogen-activated protein kinase kinase 1 [246,248]. In addition to the ERK pathway, P_i_ induces nitrate or nitrite, which stimulates nitric oxide synthase (NOS) production and, in turn, stimulates chondrocyte apoptosis [248]. Furthermore, the acute chondrocyte-specific deletion of Pit1 in mice results in pronounced cell death in the first two postnatal days, possibly owing to P_i_ transport-independent ER stress [214]. Chondrocytes might also regulate systemic P_i_ homeostasis by secreting FGF23 [244], but it is unknown whether this is under the feedback control of P_i_.

In summary, P_i_ stimulates hypertrophic differentiation and apoptosis in chondrocytes via PIT1, ERK1 and ERK2, and possibly via NOS, which is necessary for normal bone growth and possibly articular cartilage function.

### 7.3. Role of Phosphate in Osteoblasts and Osteocytes

In the vertebrate skeleton, osteoblasts and osteocytes are responsible for the synthesis of the bone matrix [249]. The bone matrix is composed of type 1 collagen, non-collagenous proteins (such as osteocalcin) and small integrin-binding ligand, N-linked glycoprotein (SIBLING) proteins [including dentin matrix acidic phosphoprotein 1 (DMP1), matrix extracellular phosphoglycoprotein (MEPE), and OPN] [250]. When osteoblasts become buried in the bone matrix, they undergo terminal differentiation into osteocytes, which serve as mechanosensors and secrete endocrine and paracrine factors to maintain skeletal homeostasis [251]. P_i_ may stimulate osteoblast proliferation and differentiation, as it induces the expression of genes important for cell proliferation, energy metabolism, and mineralization in osteoblast-like cells [47,252,253]. Similarly, P_i_ might also stimulate insulin-like growth factor 1 expression in the mouse-derived osteoblast cell line MC3T3-E1, which enhances osteoblast proliferation in an autocrine fashion [223,254]. In MC3T3 cells and primary murine calvaria-derived osteoblasts, P_i_ induces the expression of Fos-related antigen 1, *Opn*, and matrix Gla protein (which are genes required for mineralization) [223,255]. This process is dependent on ERK1 and ERK2 [223,255], further supporting the role of P_i_ in mineralization.

P_i_ stimulates osteocyte maturation and matrix formation in the osteocyte lacuna. This process can be modeled in IDG-SW3 osteocyte-like murine cells in vitro, in which 10 mM P_i_ and 10 nM calcitriol induces the gene expression of *Galnt3*, *Dmp1*, phosphate-regulating endopeptidase homolog, X-linked, ectonucleotide pyrophosphatase-phosphodiesterase family member 1, and *Mepe* [256]. Additionally, P_i_ (and calcitriol and PTH) cause osteocytes to secrete FGF23 to regulate systemic P_i_ homeostasis [257,258,259].

In summary, P_i_ stimulates the differentiation of osteoblasts and osteocytes, matrix maturation, and bone formation. These processes involve the function of P_i_ transporters and ERK1/2 signaling in vitro. The mild bone and mineral metabolism phenotypes of the global *Pit1* and *Pit2* null mice suggest a high degree of redundancy of these generally co-expressed transporters [46,260]. Bone-specific ablation of *Pit1* and *Pit2* (individually and in combination) in mice might be required to shed light on their metabolic and endocrine functions. 

### 7.4. Role of Phosphate in Osteoclasts and Bone Resorption

Osteoclasts are large, multinucleated cells derived from the monocyte lineage that are responsible for bone resorption [261], which is necessary for the remodeling and repair of the skeleton. Osteoclasts express NPT2A, PIT1, and PIT2 [262]. A concentration of 4 mM extracellular P_i_ inhibits osteoclast-like cell formation in mouse bone marrow cells [237]. This extracellular P_i_ concentration similarly decreases the number and area of resorption pits formed by mature rat osteoclasts on sperm whale dentine slices, which is a common assay for osteoclast function [263]. This observation presumably reflects a feedback mechanism to limit the degradation of hydroxyapatite. This feedback mechanism might involve the NPT2A-dependent inhibition of RANK–RANKL signaling, the inhibition of osteoclast growth by P_i_ [236], and the suppression of microRNA 223 expression (which was reported in the pre-osteoclast RAW264.7 cell line [264] and in Npt2a-null mice [265]). P_i_ reduces the gene expression of RANKL in osteoblast lineage cells, which results in the suppression of RANK in osteoclasts and the inhibition of osteoclastogenesis and bone resorption [237]. However, some P_i_ is required for normal osteoclast function. Both WT mice fed a low P_i_ diet and Hyp mice (a murine model of X-linked hypophosphatemic rickets, XLH) exhibited decreased osteoclast numbers in osteoclast-like cells derived from bone marrow cells compared with WT mice fed normal P_i_ diets [266]. This defect was reversed by a high P_i_ diet [266]. PFA, an inhibitor of Na^+^-P_i_ cotransporters, reduces bone resorption in cultured osteoclasts, possibly by inhibiting ATP production (for which uptake of extracellular P_i_ is required) [267]. Additionally, as a result of increased mitochondrial respiration, extracellular P_i_ stimulates the production of reactive oxygen species (ROS), which are signaling factors necessary for osteoclastogenesis and which stimulate bone resorption in RAW264.7 osteoclasts [268]. Furthermore, the generation of ROS increases osteoclast function and survival, which indicates that P_i_ is required for the normal function of osteoclast cells [268].

In summary, osteoclasts express NPT2A, PIT1, and PIT2 transporters. High P_i_ levels limit the survival and differentiation of osteoclasts, which might provide a mechanism of feedback inhibition during bone resorption, which is a process that releases large quantities of P_i_. However, some P_i_ seems to be required for normal osteoclast function.

## 8. Importance of Dietary Phosphorus for Teeth (or Dental Health)

The teeth are comprised of an enamel that is formed by epithelial cells (called amelobasts) and dentin [269] that is formed by mesenchymal cells (called odontoblasts); both tissues surround the dental pulp, which is a soft connective tissue that contains blood vessels and nerve fibers [269]. Cementum, periodontal ligament, and alveolar bone connect the teeth to the jaw [269]. Similar to the bone, hydroxyapatite is a major component of enamel and dentin. Different from the hydroxyapatite in the skeleton, in teeth, it is fluorinated, contains metal cation substitutions for calcium, and carbonate substitutions for P_i_ [270]. Fluorapatite (FAP, [Ca_10_(PO_4_)_6_F*_2_*]) is less soluble than hydroxyapatite [270], which explains why fluoride is an effective agent to improve dental health (though only within a narrow window of 3–4 mg for adults, whereas excessive levels lead to dental fluorosis) [270,271].

Adequate nutritional quantities of calcium and phosphorus are important for dental mineralization. A dietary ratio of 4–5 was necessary for normal bone and dentin calcification in rats that were observed from 23 to 70 days of age [272,273]. Calcium and phosphorus deficiency disturb calcification of the growing dentin and alveolar bone in rats [272,274], although enamel formation and calcification are not impaired [272]. In young rats, dietary phosphorus deficiency in the absence of vitamin D reduces overall incisor tooth mass [273,275], which is not observed in adult rats [273]. Moreover, histomorphometric evaluation shows increased levels of alveolar bone resorption in response to calcium and phosphorus deficiency [274,276].

Excessive phosphorus intake, on the other hand, also produces an inappropriate Ca/P ratio, and it was shown in degus (*Octodon degu*) to increase amelogenesis and dentinogenesis [277]. The results were a thicker enamel layer, the formation of enamel pearls, and altered dentin structure [277]. The authors also observed enamel depigmentation and hypoplasia, as well as a loss of the superficial enamel layer and a pitted appearance of the enamel layer [277]. This effect on enamel formation might occur during the secretory stage [277]. Moreover, a 1.3-fold increase in dental decay was observed in children consuming diet sodas, which can contain high concentrations of P_i_ [278]. Additionally, high phosphorus exposure infamously led to osteonecrosis of the jaw (a condition commonly known as “phossy jaw”) in 19th-century matchmakers [279]. Specifically, these workers developed gingivitis and sequestration of the alveolar crest bone, and they experienced osteonecrosis of the mandibular and maxillary bones [279]. Though “phossy jaw” is not a clinical issue in the present, there is concern that “bisphossy jaw” may be its modern-day equivalent. Oral bisphosphonates often treat disorders such as osteoporosis, but these medications carry a concerning side effect of maxillary and mandibular bone necrosis and sequestration [280]. However, the mechanism through which oral bisphosphonates cause osteonecrosis is unknown [279].

### Role of Phosphate in the Tooth

Among the currently known P_i_ transporters (Slc34a1, Slc34a2, Slc34a3, Slc20a1, Slc20a2, and Xpr1), SLC20A2/PIT2 is the most highly expressed in teeth [281]. However, knockout mouse models showed that no single transporter is essential for initiation of the mineralization process [281]. *PIT1* is expressed in ameloblasts and odontoblasts, while *PIT2* is expressed in the subodontoblastic cell layer and the stratum intermedium of ameloblasts [281,282]. PIT2 appears to be involved during the mineralization of dentin, as suggested by the dentin dysplasia described in the global *Pit2* knockout [281]. *Slc34a1/Npt2a* and *Slc34a2/Npt2b* are expressed in the MRPC-1 rat odontoblast-like mineralizing pulpal cell line [283,284]. *Slc34a2/Npt2b* is negligibly expressed in ameloblasts during the secretory stage, but it is significantly upregulated in the maturation stage [281,284,285]. However, the role of Npt2b in tooth development and mineralization is unknown [284].

Individuals with XLH (a hypophosphatemic condition characterized by FGF23 overproduction [72]) exhibit various dental abnormalities. These abnormalities include the abnormal mineralization of dentin and increased pulp chambers (resulting in dental fractures), less abundant cementum (resulting in impaired tooth attachment), and an increased risk of periodontal disease and the development of dental abscesses [21,286,287]. Enamel is largely unaffected in XLH [288]. Whether these changes are caused by hypophosphatemia alone or in conjunction with FGF23 excess remains to be shown.

## 9. Importance of Dietary Phosphorus for Cardiovascular Health

### 9.1. Role of Phosphate in Cardiac Muscle Function

Hypophosphatemia causes skeletal and cardiac myopathy by reducing intramuscular ATP synthesis and decreasing 2,3-bisphosphoglycerate (2,3-BPG) in erythrocytes (which reduces skeletal muscle oxygenation) [216,217,289,290]. Additionally, ventricular arrhythmia can occur in the context of acute myocardial infarction [291]. These hypophosphatemic effects are largely reversible but can lead to rhabdomyolysis, heart failure, and death in some cases [92,93,289,292,293,294,295].

On the other hand, hyperphosphatemia is associated with myocardial hypertrophy in rats [296] and humans with CKD [297,298,299]. High serum P_i_ is also associated with increased cardiovascular morbidity and mortality in these patients [299,300]. Finally, hyperphosphatemia can often cause vascular calcification in CKD, and the extent and histoanatomic type of calcification predict subsequent mortality [301,302].

These effects are thought to be mediated by FGF23, which causes endothelial dysfunction and increases arterial stiffness [303], activates the renin-angiotensin system [304], and causes inflammation [305], vascular calcification [306], and left ventricular hypertrophy (LVH) [307,308]. For an in-depth examination of the link between FGF23 and cardiovascular disease, see the excellent review by Stohr et al. [308]. Additionally, secondary hyperparathyroidism is associated with heart failure [309], hypertension [310], LVH [311,312], arrhythmias [312], and calcific valvular disease [311,312].

### 9.2. Role of Phosphate in Vascular Health

Hyperphosphatemia causes vascular smooth muscle cell (VSMC) apoptosis, osteogenic transdifferentiation, and vascular calcification [313,314,315]. VSMC apoptosis requires the downregulation of growth-arrest specific gene 6 and its receptor, Axl [316]. This downregulation reduces phosphatidylinositol 3-kinase-mediated phosphorylation of protein kinase B, which is also known as AKT [316]. As a result, Bcl2 (an anti-apoptotic protein) is inactivated, and Bad (a pro-apoptotic mediator) and caspase 3 are activated [316]. The osteogenic transdifferentiation of VSMCs and vascular calcification require PIT1 and PIT2 and the activation of ERK1/2 in a transport-independent manner [224,225,317]. Additionally, the activation of WNT–β-catenin–runt-related transcription factor 2 signaling (an anabolic signaling pathway important for the function of osteoblasts and osteocytes) by P_i_ can be observed [46,318,319,320,321]. Conversely, transdifferentiation is inhibited by secreted frizzled-related protein 5 [46,322]. PIT2 also mediates P_i_ uptake into the microglia, which inhibits vascular calcification in the basal ganglia in an interplay with the P_i_ exporter XPR1 [187,323]. Additional sodium-independent transport systems for the intake and efflux of P_i_ may exist in VSMCs, which have not been well-characterized [198,324,325].

High dietary phosphorus finally reduces endothelium-dependent vasodilation *in vitro* and was shown to reduce flow-mediated vasodilation in healthy men [326]. In a study of normal U.S. adults, Kendrick et al. showed that high-normal levels of serum P_i_ are associated with a high ankle-brachial pressure index, which is a marker for arterial stiffness [327]. Thereby, high dietary phosphorus may acutely increase the risk of cardiovascular mortality [326].

### 9.3. Role of Phosphate in Erythrocyte Function

P_i_ affects erythrocyte function directly [289,290] and indirectly via FGF23 [328]. Hypophosphatemia reduces the concentration of 2,3-BPG in erythrocytes, since P_i_ is required for the synthesis of ATP and thus for the glycolytic synthesis of the 2,3-BPG precursor, 1,3-bisphosphoglycerate [290]. For example, Lichtman et al. found a 45% reduction of 2,3-BPG in the erythrocytes of patients with parenteral nutrition-induced hypophosphatemia [290]. Reduced concentrations of 2,3-BPG shift the oxyhemoglobin dissociation curve to the left (increasing hemoglobin affinity for O_2_), and thereby hypophosphatemia can cause tissue hypoxia [289].

Blood P_i_ may indirectly affect hematopoesis by regulating FGF23. FGF23 may stimulate hematopoiesis, as suggested by low erythrocyte counts found in FGF23 null mice [329]. In turn, erythropoietin may stimulate the synthesis and secretion of FGF23 by myeloid lineage LSK cells in the hematopoietic bone marrow [330].

## 10. Importance of Dietary Phosphorus for Skeletal Muscle Health

Similar to cardiac muscle, P_i_ is essential in skeletal muscle as a substrate for ATP and CrP synthesis [331,332]. Hypophosphatemia causes a reduction in ATP flux (V_ATP_) in mouse models [217]. Similarly, the ablation of *Pit1* and *Pit2* in mice is post-natally lethal due to a generalized skeletal muscle myopathy [333]. Likewise, patients with hypophosphatemia develop myopathy in addition to rickets and osteomalacia [332]. Moreover, iatrogenic P_i_ depletion in patients with chronic renal failure results in proximal myopathy [8], and rhabdomyolysis can occur with severe hypophosphatemia superimposed on simple phosphorus deficiency [92,292,334].

On the other hand, hyperphosphatemia may contribute to the development of muscle weakness and frailty, at least in patients with CKD [335,336]. High-medium P_i_ concentrations cause protein loss in myotubes from rat L6 cells and stimulate autophagy, resulting in myotube atrophy [337].

### Role of Phosphate in Skeletal Muscle

We recently showed that hypophosphatemia causes a reduction in V_ATP_, as shown by a 50% reduction in WT mice fed a low P_i_ diet [217]. V_ATP_ in these mice normalized after intravenous P_i_ supplementation [217]. Likewise, the simultaneous conditional deletion of all four Pit1/2 alleles in mouse skeletal muscle causes muscular atrophy and myofiber degeneration by post-natal day 10 (P10) [333]. These mice die by P13 [333]. Similarly, the ablation of three of four Pit1/2 alleles in skeletal muscle reduced the running ability of these mice as measured by wheel turns/day [333]. This result suggested that the loss of Pit1/2 causes impaired muscle function [333]. Studies in C2C12 myocyte cultures suggest that P_i_ acts at complex V of the respiratory chain as a substrate for ATP synthesis during oxidative phosphorylation [216,217]. 

Little is known so far about the molecular mechanism, but P_i_ appears to maintain cytochrome b oxidation and cytochrome c reduction [338] and to stimulate the activity of several Krebs cycle dehydrogenases: 2-oxoglutarate dehydrogenase, isocitrate dehydrogenase, and malate dehydrogenase [339,340,341,342]. This action of P_i_ increases the concentrations of mitochondrial electron donors (FADH, NADH, and NADPH) to fuel the electron transport chain. Additionally, P_i_ is an important cofactor for glyceraldehyde 3-phosphate dehydrogenase, an important rate-limiting glycolytic enzyme that generates NADH [343,344].

The type III Na^+^/P_i_ cotransporters PIT1 and PIT2 mediate P_i_ uptake into the sarcoplasma [345,346,347,348], and it is then transported into the mitochondria by PIC [21] and the mitochondrial dicarboxylate carrier [349,350]. PIC is also a component of the mPTP, which regulates calcium transport into the mitochondrial matrix [213]. Human LOF mutations of *SLC25A3* [183,184,213,351] or cardiac-specific deletion in mice [352] cause cardiomyopathy.

However, the mechanism whereby blood P_i_, PIT1, and PIT2 modify muscle ATP synthesis is not clear, since the hydrolysis of ATP and CrP during exercise may generate sufficient P_i_ inside myofibers for ATP synthesis. In fact, the sarcoplasmic buildup of P_i_ may be an important cause of muscle fatigue [353]. Sarcoplasmic P_i_ can increase from 5–30 mM following intense exercise, which has been shown to reduce peak force by decreasing force per actin-myosin bridge, by increasing the number of low-force bridges in skeletal muscle, by decreasing cytosolic ionized calcium, and by causing calcium–P_i_ precipitations in the sarcoplasmic reticulum [46,353,354,355,356,357].

Since the activation of the ERK1/2 pathway [194,204] is necessary for normal neuromuscular junctions [358,359], therefore, an intriguing possibility is that the transport-independent signaling functions of PIT1/2 are important for skeletal muscle function [204,333].

In summary, P_i_ is required to maintain muscle function, but excess P_i_ leads to calcification (which is best documented in vascular smooth musculature) and skeletal muscle fatigue. This process may involve the functions of the PIT1/2 transporters and of ERK1/2 signaling.

## 11. Importance of Dietary Phosphorus for Healthy Aging

As examined in the previous chapters of this review, concentrations of P_i_ that are either too high or too low both have detrimental health effects. Since P_i_ depletion in terrestrial animals and humans is rare due to the ubiquitous nature of phosphorus in the diet (particularly in processed foods [96] and when used in high doses to treat hypophosphatemic disorders [360]), we will focus our discussion of longevity on the effects of high dietary phosphorus.

### 11.1. High Dietary Phosphate Reduces Longevity in Lower Species

We previously used *Drosophila melanogaster* to study the effects of high dietary P_i_ on longevity. Supplementation of standard medium (SM) with 30 mM P_i_ reduced fly lifespan to 38 ± 2.4 days [361]. In contrast, control *yw* flies had a median lifespan of 42 ± 0.8 days, and flies cultured in SM + 30 mM sodium sulfate had a median lifespan of 44 ± 0.8 days [361]. Median lifespan increased to 49 ± 1.9 days and 47 ± 1.8 days, respectively, by inhibiting P_i_ intake through the addition of either sevelamer (which blocks P_i_ absorption) or PFA (which inhibits the cellular uptake of P_i_) to the SM [361]. Finally, more recent findings show that PFA and the ablation of MFS2 (a P_i_ transporter in fly Malphigian tubules that functions in P_i_ excretion) increase blood P_i_, decrease the formation of Malphigian tubule calcium-P_i_ stones, and increase lifespan in *Drosophila* [362]. These results suggest that the excretion of P_i_ and the formation of Malpighian tubule stones reduce the longevity of flies cultured on a high P_i_ medium [362].

### 11.2. High Dietary Phosphorus Reduces Longevity in Higher Species and Humans

Similarly, in higher species such as mice, high dietary phosphorus negatively affects longevity. P_i_ loading in uremic rats dose-dependently induces inflammation in the aorta, heart, and kidneys [363]. Furthermore, *Klotho* null mice have severe hyperphosphatemia [364,365]. These mice die prematurely due to vascular and renal calcification, as well as atrophy of the skin, muscle, intestinal, and gonadal tissues [364,365].

The aging-like syndrome of *Klotho^−/−^* mice is ameliorated when serum P_i_ levels are normalized in *Npt2a^−/−^* and *Klotho^−/−^* double-knockout mice, and it is induced again by placing these double-knockout mice on a high P_i_ diet [364,365]. This dietary P_i_ toxicity in *Npt2a^−/−^* and *Klotho^−/−^* double-knockout mice very much resembles the potential of dietary P_i_ to modify mortality in CKD patients, as discussed above [363,366,367,368].

In addition, P_i_ has recently been implicated in cancer aggressiveness [369]. *SLC20A1* may be overexpressed in tongue tumors [370,371], and Npt2b expression is increased in lung cancers [369,371]. Furthermore, high dietary P_i_ stimulates the AKT-mammalian target of rapamycin regulatory pathway, leading to higher lung cancer aggressiveness in K-*ras*^LA1^ mice [369,371]. The knockdown of Npt2b with siRNA was shown to decrease the number and size of lung tumors in this mouse model, suggesting that the regulation of P_i_ consumption through NPT2b knockdown may be a possible treatment for lung cancer [371,372]. Similarly, a high P_i_ concentration in the tumor microenvironment has been identified as a marker for tumor progression in mouse mammary gland tumors [371,373]. For a more detailed overview of the role of P_i_ transporters in cancer and tumor biology, refer to the excellent review by Lacerda-Abreu et al. [371].

Finally, high dietary phosphorus increases fracture risk [241], which has implications for lifespan, since excess mortality for five years following a proximal non-hip or lower leg fragility fracture, and ≥10 years following a hip fracture, have been reported [374]. These results together further support a role for excess phosphorus in reducing longevity, even in humans who have normal kidney function.

## 12. Conclusions

Phosphorus is an important nutrient in our diet. P_i_ serves crucial functions as an important physiologic buffer, as a substrate for critical cellular functions, and along with calcium as a component of the bone mineral in the skeleton. P_i_ is absorbed in the small intestine via a paracellular route of passive diffusion or a transcellular route via P_i_ transporters such as NPT2b. Interruption of these absorption processes can result in hypophosphatemia. Since phosphorus has become abundant in Western diets—where it often supplements prepared foods—it is also important to consider that excess levels of dietary phosphorus have adverse health effects. High dietary phosphorus has been implicated in several processes related to accelerated aging: for instance, increased fracture risks, cancer proliferation, cardiac and skeletal muscle dysfunction, and vascular calcification. Currently, it is estimated that dietary phosphorus exceeds the RDA by 1.5–2X, which is of particular concern for individuals with cardiovascular diseases or CKD. A better declaration and regulation of P_i_ additives in the processed foods industries, and more clinical research, is clearly needed to understand how to optimize nutritional phosphorus best while avoiding toxic side effects. Of particular interest for the latter are agents that target NPT2b to block intestinal P_i_ absorption, agents that regulate PIT1/2 (and possibly IP7/IP8/XPR1) to optimize cellular P_i_ homeostasis, and agents that improve renal P_i_ excretion via NPT2a and NPT2c. Furthermore, the contributions of type I (SLC17) and type III (SLC20) P_i_ transporters, and the molecular basis for paracellular diffusion in the intestine and proximal renal tubules, are still largely unknown. Thus, research into these pathways could provide novel targets for the development of therapeutics that improve the outcomes of individuals with cardiovascular diseases or CKD.

### Key Points

Phosphorus is abundant in Western diets, and understanding endocrine and metabolic P_i_ sensing is essential to understand human disorders. Both high and low dietary phosphorus can cause adverse health effects and impair longevity, and it may be important to consider implementing phosphorus analysis as a routine measurement in clinical practice. Declaration of P_i_ additive use by the food industry may be helpful, since currently dietary phosphorus intake routinely exceeds the RDA.

## Figures and Tables

**Table 1:** Clinician’s Guide to P_i_/Vitamin D Supplementation ^1^.

Phosphate Preparations	Phosphorus Content	Potassium (K) Content	Sodium (Na) Content
Neutraphos-powder (for mixing with liquid)	250 mg/packet	270 mg/packet	164 mg/packet
Neutraphos-K-powder (for mixing with liquid)	250 mg/packet	556 mg/packet	0 mg/packet
K-Phos Original-tablet (to mix in liquid, acidifying)	114 mg/tablet	144 mg/tablet	0 mg/tablet
K-Phos MF-tablet (mixing not required, acidifying)	126 mg/tablet	45 mg/tablet	67 mg/tablet
K-Phos #2 (double strength of K-Phos MF)	250 mg/tablet	90 mg/tablet	133 mg/tablet
K-Phos Neutral-tablet (non-acidifying, mixing not required)	250 mg/tablet	45 mg/tablet	298 mg/tablet
Phospha-Soda-solution (small doses may be given undiluted)	127 mg/mL	0 mg/mL	152 mg/mL
Joulie’s solution (prepared by compounding pharmacies)	30 mg/mL	0 mg/mL	17.5–20 mg/mL
**Vitamin D and Related Agents**	**Agent**		**Available Preparations**
Vitamin D	Calciferol (Drisdol)		Solution: 8000 IU/mL Tablets: 25,000 and 50,000 IU
Dihydrotachysterol	DHT (Hytakerol)		Solution: 0.2 µg/5 mL Tablets: 0.125, 0.2, and 0.4 mg
1,25 dihydroxyvitamin D	Calcitriol (Rocaltrol)		0.25 and 0.5 µg capsules and 1 µg/mL solution
	Calcijex		Ampules for IV use containing 1 or 2 µg of drug per mL
1α-hydroxyvitamin D	Alfacalcidol		0.25, 0.5, and 1 µg capsules Oral solution (drops): 2 µg/mL Solution for IV use: 2 µg/mL
Vitamin D analogs	Paricalcitol (Zemplar)		1, 2, and 4 µg capsules
	Doxercalciferol (Hectoral)		0.5, 1, and 2.5 µg capsules

^1^ SI conversion: 1 mg phosphorus = 0.32 mmol phosphorus, 1 µg vitamin D  =  40 IU vitamin D. IU, international unit. IV, intravenous. From: [102].

**Table 2:** Acquired Disorders of Phosphate Homeostasis ^1^.

Disorder	Mechanism	Ref.
**Hypophosphatemic**		
Dietary phosphorus deficiency	Total body deficiency, combined with an insulin-mediated cellular shift in P_i_ during refeeding, causes hypophosphatemia.	[50]
Vitamin D deficiency	Reduced intestinal Ca and P_i_ absorption causes rickets/osteomalacia and secondary hyperparathyroidism.	[130,131]
Chronic use of P_i_ antacids/high gastric pH (due to PPIs, autoimmune gastritis/pernicious anemia, etc.)	High gastric pH reduces P_i_ solubility, which potentially results in reduced mineral absorption and hypophosphatemia.	[109,111,112,132,133]
Reduced gastrointestinal absorption (due to Inflammatory Bowel and Celiac diseases, diarrhea, vomiting, short gut, intestinal mucosal hypoplasia, jejunal feeding, prematurity, etc.)	Chronic diarrhea and reduced gastrointestinal absorption of P_i_ reduce bioavailable P_i_.	[127,128]
Parenteral iron administration	Ferric carboxymaltose blocks FGF23 cleavage, which induces renal P_i_ wasting.	[134]
Proximal tubular damage (caused by renal tubular acidosis or drugs such as theophylline, foscarnet)	Renal Pi wasting causes rickets/osteomalacia and hypercalciuria.	[135,136,137]
Hyperparathyroidism	Bone resorption increases serum P_i_, but the net effect is to lower serum P_i_ due to increased renal excretion.	[138,139]
**Drugs**		
Phosphonocarboxylates (e.g., PFA), phloretin derivatives (e.g., 2′-PP), arsenate	Competitively inhibit Na-P_i_ co-transport of P_i_.	[115,116,118,140]
Niacin/Nicotinamide, NAD, triazole derivatives	Downregulates NPT2b, inhibits intestinal P_i_ transport.	[117,118,141]
Tenapanor	Inhibits paracellular P_i_ transport and downregulates NPT2b.	[118,120]
Insulin	Promotes P_i_ uptake into tissues. Can result in hypophosphatemia in the context of refeeding.	[103,142]
Bisphosphonates and other bone resorption blockers	Decreased bone resorption can cause hypophosphatemia along with hypocalcemia.	[143,144]
Adriamycin	Inhibits P_i_ transport by PIC in reconstituted liposomes.	[145]
**Hyperphosphatemic**		
High phytate/low Ca^2+^ diet	Low dietary Ca^2+^ causes P_i_ hyperabsorption. The associated homeostatic response induces secondary hyperparathyroidism.	[113]
Tumor lysis syndrome and rhabdomyolysis	Release of intracellular P_i_ from lysed cells may result in hyperphosphatemia.	[146,147]
Bone metastases	Tumor metastasis can increase bone resorption, which may result in hyperphosphatemia and hypercalcemia.	[148]
Kidney failure (e.g., CKD)	Reduced number of nephrons decreases renal P_i_ excretion, resulting in hyperphosphatemia.	[149]
Lowered gastric pH	May increase P_i_ bioaccessibility and P_i_ absorption.	[107,108,110]
**Drugs**		
Vitamin D	Increases intestinal absorption of Ca and P_i_, increases bone resorption, suppresses PTH, and thereby reduces renal excretion of P_i_, all of which contribute to hyperphosphatemia.	[7,54,55,66,124]
P_i_ supplementation	P_i_-containing laxatives can induce severe hyperphosphatemia, nephrocalcinosis, and renal failure.	[105,106]
Pharmaceutical agents increase serum P_i_	Refer to Table 1.	
FGFR Inhibitors	Inhibit renal FGF23 signaling.	[150]

^1^ Table modified from [46]. Gray background color was implemented to provide a visual boundary between hypophosphatemic and hyperphosphatemic categorization. Bold font was used to visually denote the boundary between drug-related and other disorders. Ca/Ca^2+^, calcium. P_i_, inorganic phosphate. Na, sodium. PPI, proton pump inhibitor. FGF23, fibroblast growth factor 23. PFA, phosphonoformic acid. 2′-PP, 2′-phosphophloretin. NAD, nicotinamide adenine dinucleotide. NPT2b, type IIB sodium-dependent phosphate cotransporter. PIC, mitochondrial phosphate carrier. CKD, chronic kidney disease. PTH, parathyroid hormone. FGFR, fibroblast growth factor receptor.

**Table 3:** Human Genetic Disorders of P_i_ Homeostasis ^1^.

Disorder	Abbreviation	Inheritance	Gene	Mechanism	Ref.
**Hyperphosphatemic Disorders**					
Hyperphosphatemic Familial Tumoral Calcinosis type 1 and the allelic variant Hyperostosis–Hyperphosphatemia Syndrome	HFTC1 HSS	AR AR	*GALNT3*	FGF23 deficiency	[156,157]
Hyperphosphatemic Familial Tumoral Calcinosis Type 2	HFTC2	AR	*FGF23*	FGF23 deficiency	[158,159]
Hyperphosphatemic Familial Tumoral Calcinosis Type 3	HFTC3	AR	*KL*	FGF23 resistance	[160]
Idiopathic Hyperphosphatasia (Juvenile Paget’s Disease)	N/A	AR	*TNFRSF11B*	OPG deficiency	[161]
Pseudohypoparathyroidism	PHP1A PHP1B	AD AD (impr.)	*GNAS* *GNAS* or up-stream regulatory region	PTH resistance, FGF23-independent	[162,163]
Familial Isolated Hypoparathyroidism	FIH	AD or AR	*CASR* *GCMB* *PTH*	PTH deficiency, FGF23-independent	[164,165,166]
Blomstrand disease	BOCD	AR	*PTHR1*	PTH resistance, FGF23-independent	[167,168]
**Hypophosphatemic Disorders**					
X-linked hypophosphatemia	XLH	X-linked	*PHEX*	FGF23-dependent	[169]
Autosomal Dominant Hypophosphatemic Rickets	ADHR	AD	*FGF23*	FGF23-dependent	[170]
Autosomal Dominant Hypophosphatemic Rickets	ADHR	AD	*KL*	FGF23-dependent	[171]
Autosomal Recessive Hypophosphatemic Rickets types 1, 2, and 3	ARHR1 ARHR2 ARHR3	AR	*DMP1* *ENPP1* *FAM20C*	FGF23-dependent	[172,173,174]
Hereditary Hypophosphatemic Rickets with Hypercalciuria	HHRH	AR	*SLC34A3*	Proximal tubular P_i_ wasting, FGF23-independent	[175,176]
Vitamin D-resistant rickets type 1A	VDDR1A	AR	*CYP27B1*	1,25(OH)_2_D deficiency, FGF23-independent	[154,177]
Hereditary 1,25(OH)_2_D-resistant rickets	HVDDR	AR	*VDR*	1,25(OH)_2_D resistance, FGF23-independent	[153,178]
Familial hypocalciuric hypercalcemia/neonatal severe hyperparathyroidism	FHH NSHPT	AD/AR	*CASR*	PTH excess, FGF23-independent	[179]
Jansen disease		AD	*PTHR1*	Const. active PTHR1, FGF23-dependent	[180,181]
**Normophosphatemic disorders**					
Pulmonary alveolar microlithiasis	PAM	AR	*SLC34A2*	Reduced alveolar epithelial P_i_ uptake	[35]
Normophosphatemic familial tumoral calcinosis	NFTC	AR	*SAMD9*	Unknown	[182]
Muscle dystrophy and cardiomyopathy	MDC	AR	*SLC25A3*	Reduced mitochondrial P_i_ uptake	[183,184]
Primary familial basal ganglial calcification type 1	PFBC1 or IBGC1	AD	*PIT2*	Reduced microglial P_i_ uptake	[185]
Primary familial basal ganglial calcification type 4	PFBC4 or IBGC4	AD	*PDGFRB*	Reduced PIT2 expression	[186]
Primary familial basal ganglial calcification type 5	PFBC5 or IBGC5	AD	*PDGFB*	Reduced PIT2 expression	[186]
Primary familial basal ganglial calcification type 6	PFBC6 or IBGC6	AD	*XPR1*	Reduced vascular P_i_ export	[187]
Primary familial basal ganglial calcification type 7	PFBC7 or IBGC7	AR	*MYORG*	Unclear, astrocyte dysfunction and possible NVU disruption may be causative factors.	[188,189]
Primary familial basal ganglial calcification type 8	PFBC8 or IBGC8	AR	*JAM2*	Reduced JAM2 expression	[190,191]

^1^ Adapted from [21]. Bold font was used to visually denote the boundaries between hyperphosphatemic, hypophosphatemic and normophosphatemic disorder categories. AD, autosomal dominant. AR, autosomal recessive. *GALNT3*, polypeptide N-acetylgalactosaminyltransferase 3. *FGF23*/FGF23, fibroblast growth factor 23. *KL*, klotho. *TNFRSF11B*, TNF receptor superfamily member 11B. *GNAS*, guanine nucleotide- binding protein, alpha stimulating. *CASR*, calcium-sensing receptor. *GCMB*, glial cell missing gene. *PTH*/PTH, parathyroid hormone. *PTHR1*/PTHR1, parathyroid hormone 1 receptor. *PHEX*, phosphate-regulating endopeptidase homolog, X-linked. *DMP1*, dentin matrix acidic phosphoprotein 1. *ENPP1*, ectonucleotide pyrophosphatase-phosphodiesterase family member 1. *FAM20C*, golgi-associated secretory pathway kinase. *SLC34A3*, solute carrier family 34 member 3. *CYP27B1*, vitamin D 1-α hydroxylase. *VDR*, vitamin D receptor. *SLC34A2*, solute carrier family 34 member 2. *SAMD9*, sterile alpha motif domain containing 9. *SLC25A3*, solute carrier family 25 member 3. *PIT2*/PIT2, type III sodium-dependent phosphate transporter 2. *PDGFRB*, platelet derived growth factor receptor beta. *PDGFB*, platelet derived growth factor subunit B. *XPR1*, xenotropic and polytropic retrovirus receptor 1. *MYORG*, myogenesis regulating glycosidase. *JAM2*/JAM2, junctional adhesion molecule 2. OPG, osteoprotegerin. P_i_, inorganic phosphate. 1,25(OH)_2_D, 1,25-dihydroxyvitamin D. NVU, neurovascular unit.

## Glossary

**Key Term/Abbreviation**	**Definition**
(I, II, III, IV, V)	Complexes I-V of the mitochondrial respiratory chain
1,25-dihydroxyvitamin D	1,25(OH)_2_D
2′-PP	2′-Phosphophloretin
2,3-BPG	2,3-Bisphosphoglycerate
AD	Autosomal dominant
AKT	Protein kinase B
Alp	Alkaline phosphatase
AMP	Adenosine monophosphate
AMPK	AMP-activated protein kinase
AR	Autosomal recessive
ATP	Adenosine triphosphate
BBMV	Brush border membrane vesicle
BPG	Bisphosphoglycerate
CASR	Calcium-sensing receptor
CKD	Chronic kidney disease
c-myb	V-myb avian myeloblastosis viral oncogene homolog
CrP	Creatine phosphate
CYP24A1	The vitamin D 24-hydroxylase
CYP27B1	The vitamin D 1-α hydroxylase
DMP1	Dentin matrix acidic phosphoprotein 1
DNA	Deoxyribonucleic acid
EGF	Epidermal growth factor
EGR1	Early growth response 1
Enpp1	Ectonucleotide pyrophosphatase-phosphodiesterase family member 1
ER	Endoplasmic reticulum
ERK1	Extracellular signal-regulated kinase 1
ERK2	Extracellular signal-regulated kinase 2
Etv5	ETS variant 5
FADH	Flavin adenine dinucleotide
FAM20c	Golgi-associated secretory pathway kinase
FAP	Fluorapatite mineral
FGF	Fibroblast growth factor
FGF23	Fibroblast growth factor 23
FGFR1	Fibroblast growth factor receptor 1
FGFR1c	FGFR1 isoform c
FRS2	FGFR substrate 2
GALNT3	Polypeptide N-Acetylgalactosaminyltransferase 3
GC	Glucocorticoid
GCMB	Glial cell missing gene
GI	Gastrointestinal
GNAS	Guanine nucleotide-binding protein, alpha stimulating
GRB2	Growth factor receptor bound protein 2
HVDDR	Hereditary 1,25(OH)_2_D-resistant rickets
IBGC	Idiopathic basal ganglia calcification
IC	Intracellular
ICF	Intracellular fluid
IU	International units
IP6K1	Inositol hexakisphosphate kinase 1
IP6K2	Inositol hexakisphosphate kinase 2
IP6	Inositol hexakisphosphate
IP7	5-diphosphoinositol 1,2,3,4,6-pentakisphosphate
IP8	1,5-bisdiphosphoinositol 1,2,3,4-tetrakisphosphate
IV	Intravenous
JAM2	Junctional adhesion molecule 2
KL	α-Klotho
K_m_	Michaelis-Menten affinity constant
LOF	Loss of function
LVH	Left ventricular hypertrophy
MAPK	Mitogen-activated protein kinase
MAPK1	Mitogen-activated protein kinase 1
MAPK3	Mitogen-activated protein kinase 3
MEPE	Matrix extracellular phosphoglycoprotein
mPTP	Mitochondrial permeability transition pore
MV	Matrix vesicle
MYORG	Myogenesis regulating glycosidase
Na^+^	Sodium ion
NAD	Nicotinamide adenine dinucleotide
NADH	Reduced nicotinamide adenine dinucleotide
NADPH	Reduced nicotinamide adenine dinucleotide phosphate
NF-κB	Nuclear factor kappa-light-chain-enhancer of activated B cells
NHE3	Sodium/hydrogen exchanger isoform 3
NHERF-1	Na^+^/H^+^ exchanger regulatory factor
NOS	Nitric oxide synthase
NPT1	Sodium-dependent phosphate transport protein 1
NPT2a	Sodium-dependent phosphate transport protein 2a
NPT2b	Sodium-dependent phosphate transport protein 2b
NPT2c	Sodium-dependent phosphate transport protein 2c
NVU	Neurovascular unit
OPG	Osteoprotegerin
OPN	Osteopontin
P10	Postnatal day 10
PDGFB	Platelet-derived growth factor subunit B
PDGFRB	Platelet-derived growth factor receptor beta
PFA	Phosphonoformate/phosphonoformic acid
PFBC	Primary familial brain calcification
Phex	Phosphate-regulating endopeptidase homolog, X-linked
PHOSPHO1	Phosphoethanolamine/phosphocholine phosphatase 1
P_i_	Inorganic phosphate
PIC	Mitochondrial phosphate carrier
PIT1	Type III sodium-dependent phosphate transporter 1
PIT2	Type III sodium-dependent phosphate transporter 2
PKA	Protein kinase A
PKC	Protein kinase C
PLCγ	Phospholipase C gamma isoform.
PP_i_	Pyrophosphate
PPIs	Proton pump inhibitors
PTH	Parathyroid hormone
PTHR1	Parathyroid hormone 1 receptor
RANK	Receptor activator of NF-κB
RANKL	Receptor activator of NF-κB ligand
RDA	Recommended daily allowance
RNA	Ribonucleic acid
ROS	Reactive oxygen species
RXR	Retinoic acid X-receptor
SAMD9	Sterile alpha motif domain containing 9
SIBLING	Small integrin-binding ligand, N-linked glycoprotein
SLC17	Solute carrier family 17
SLC20A1	Solute carrier family 20 member 1; gene encoding PIT1
SLC20A2	Solute carrier family 20 member 2; gene encoding PIT2
SLC25A3	Solute carrier family 25 member 3; gene encoding PIC
SLC34A1	Solute carrier family 34 member 3; gene encoding NPT2a
SLC34A2	Solute carrier family 34 member 2; gene encoding NPT2b
SLC34A3	Solute carrier family 34 member 3; gene encoding NPT2c
SM	Standard medium
SOS	Son of sevenless
SPX	A protein domain named after SYG1/Pho81/XPR1 proteins
TEER	Transepithelial electrical resistance
TNAP	Tissue non-specific alkaline phosphatase
TNFRSF11B	TNF receptor superfamily member 11B
V_ATP_	ATP flux
VDR	Vitamin D receptor
VRE	Vitamin D-reponsive elements
VSMC	Vascular smooth muscle cell
WNT	Wingless-related integration site
WT	Wild-type
XLH	X-linked hyperphosphatemia
XPR1	Xenotropic and polytropic retrovirus receptor 1
